# Supplementary material for: Binding of Ca2+ Ions to Alkylbenzene Sulfonates: Micelle Formation, Second Critical Concentration and Precipitation
Source: Materials (Basel). 2023 Jan 4;16(2):494. doi: 10.3390/ma16020494 (PMC9864979; doi:10.3390/ma16020494)
Supplement: Supplementary file 1 [file materials-16-00494-s001.zip › materials-2065398-supplementary.pdf]

SUPPORTING INFORMATION TO

Binding of Ca^{2+} Ions to Alkylbenzene Sulfonates: Micelle Formation, Second Critical Concentration and Precipitation

Adél Anna Ádám[1], Szilveszter Ziegenheim[1], László Janovák[2], Márton Szabados[1], Csaba Bús[1], Ákos Kukovecz[3], Zoltán Kónya[3], Imre Dékány[2], Pál Sipos[4,*], Bence Kutus[4,*]

[1] Department of Organic Chemistry, University of Szeged, Dóm tér 8, 6720 Szeged, Hungary

[2] Department of Physical Chemistry and Materials Science, University of Szeged, Rerrich Béla tér 1, 6720 Szeged, Hungary

[3] Department of Applied and Environmental Chemistry, University of Szeged, Rerrich Béla tér 1, 6720 Szeged, Hungary

[4] Department of Inorganic and Analytical Chemistry, University of Szeged, Dóm tér 7, 6720 Szeged, Hungary

Correspondence: Prof. Dr. Pál Sipos, sipos@chem.u-szeged.hu

Dr. Bence Kutus, kutusb@chem.u-szeged.hu

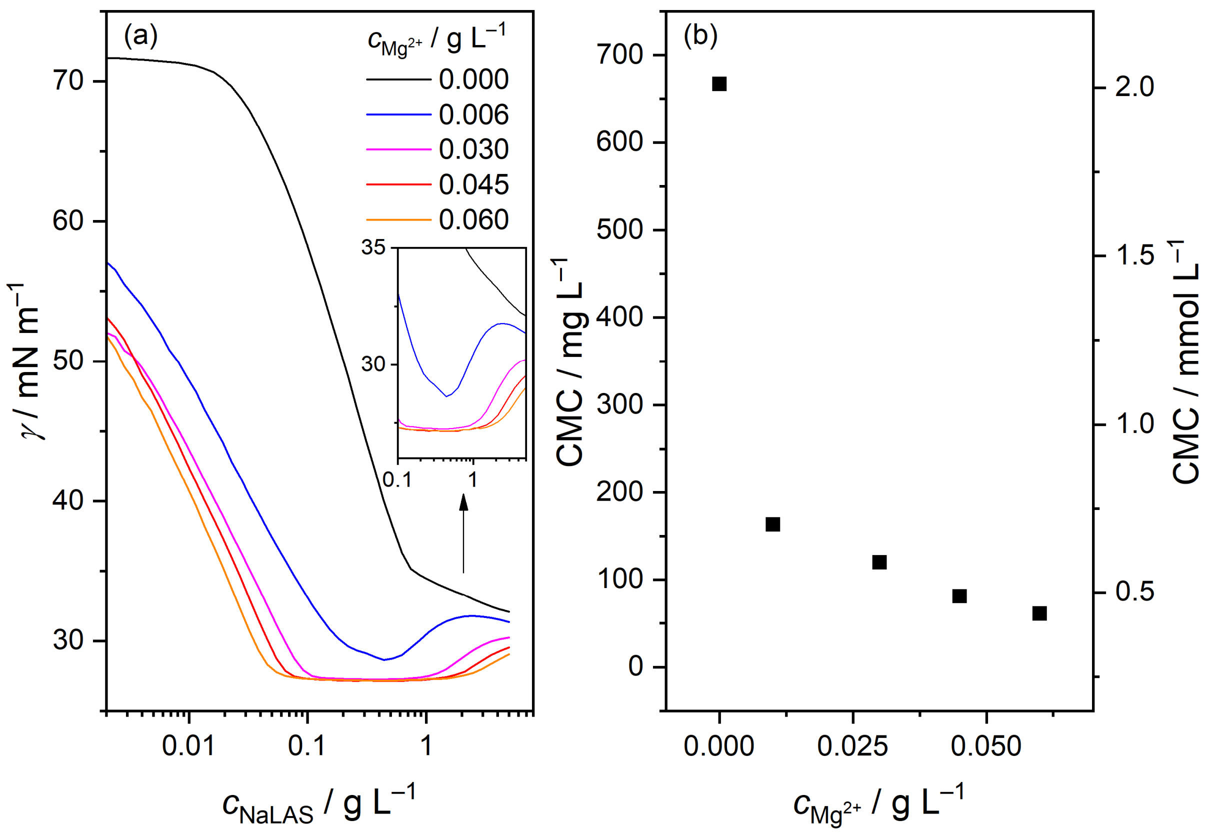

Figure S1 (a) Surface tension (γ) as a function of sodium linear alkylbenzene sulfonate (NaLAS) concentration, in the presence of $MgCl_2$. Inset: zoomed region showing the onset of the increase in γ; (b) Critical micelle concentration (CMC) in mg L^{-1} (left axis) and in mmol L^{-1} (right axis), as a function of Mg^{2+}-ion concentration. Experimental conditions: $c_{Mg^{2+}}$ = 0–0.0 g L^{-1}, c_{NaLAS} = 0.002–5.00 g L^{-1}, T = (25.0±0.1)°C.

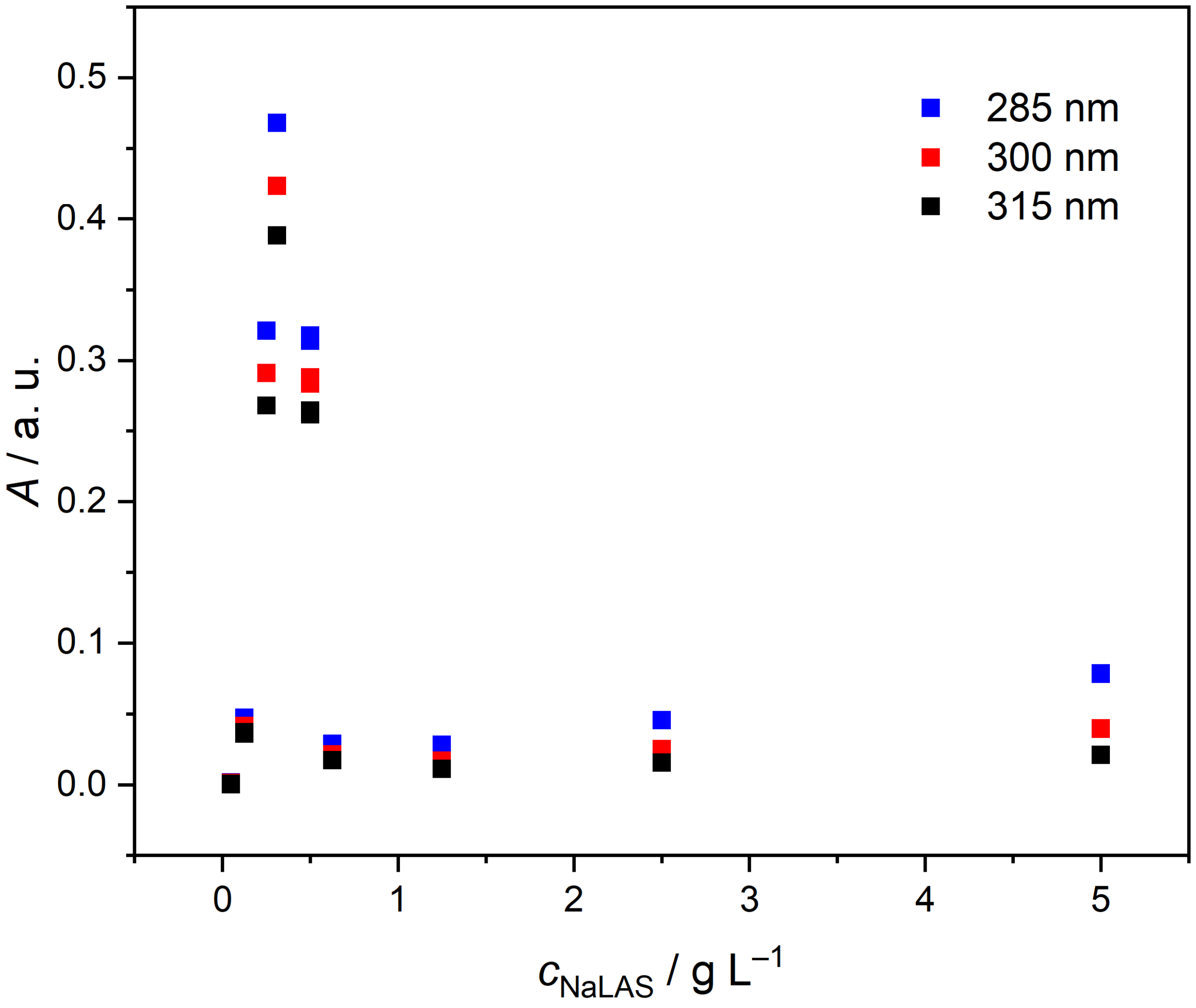

Figure S2 Apparent absorbance (A) at three different wavelengths as a function of sodium linear alkylbenzene sulfonate (NaLAS) concentration, in the presence of $CaCl_2$. Here, the absorbance measured arise from both light absorption and scattering; values close to zero indicate a transparent sample. Experimental conditions: $c_{Ca^{2+}}$ = 0–0.1 g L^{-1}, c_{NaLAS} = 0.001–5.00 g L^{-1}, T = (25±0.1) °C.

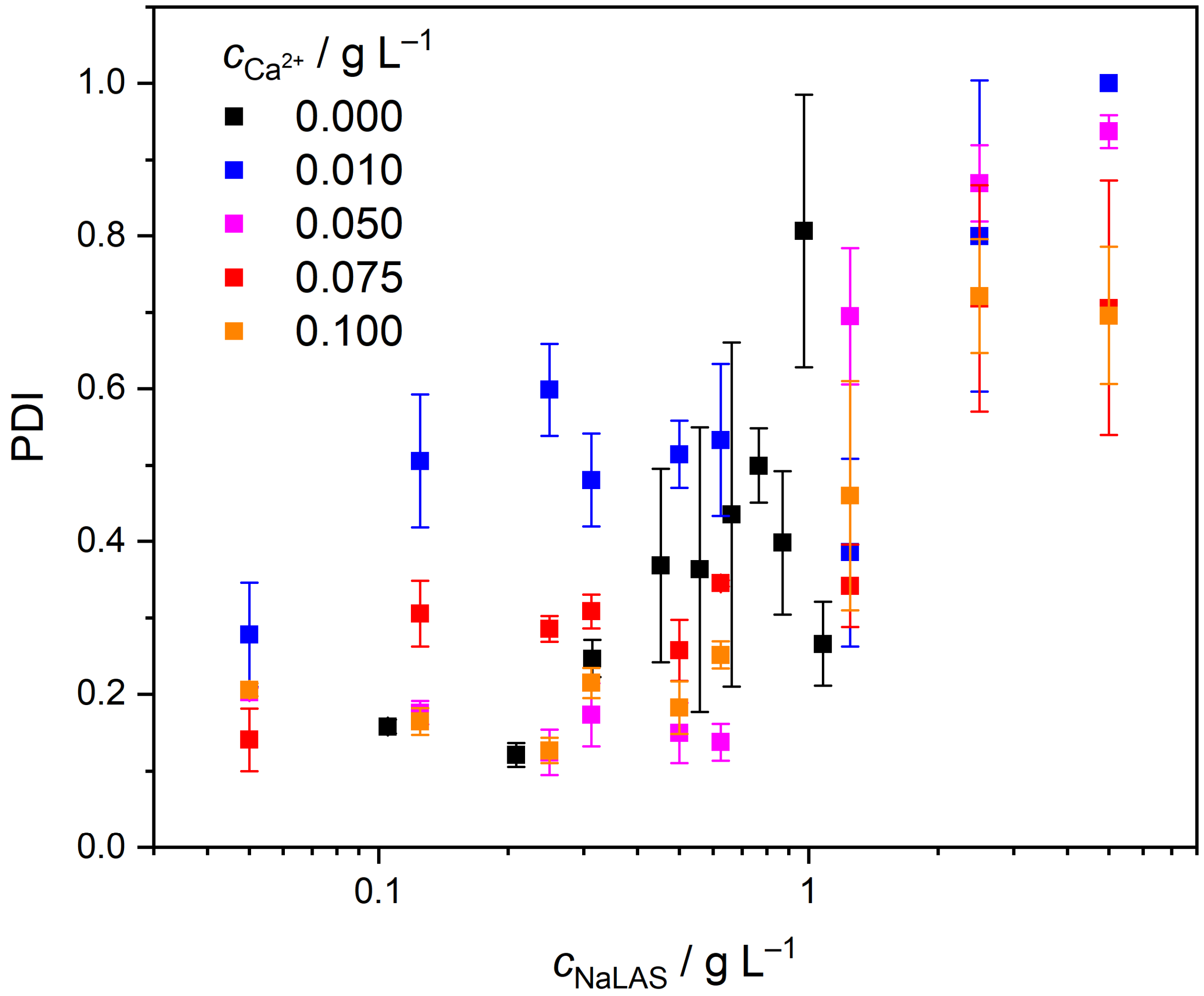

Figure S3 Polydispersity index (PDI) as a function of sodium linear alkylbenzene sulfonate (NaLAS) concentration, in the presence of $CaCl_2$. Here, the error bars represent the standard deviation of at least three values obtained for each sample. Experimental conditions: $c_{Ca^{2+}}$ = 0–0.1 g L^{-1}, c_{NaLAS} = 0.001–5.00 g L^{-1}, T = (25±0.1) °C.

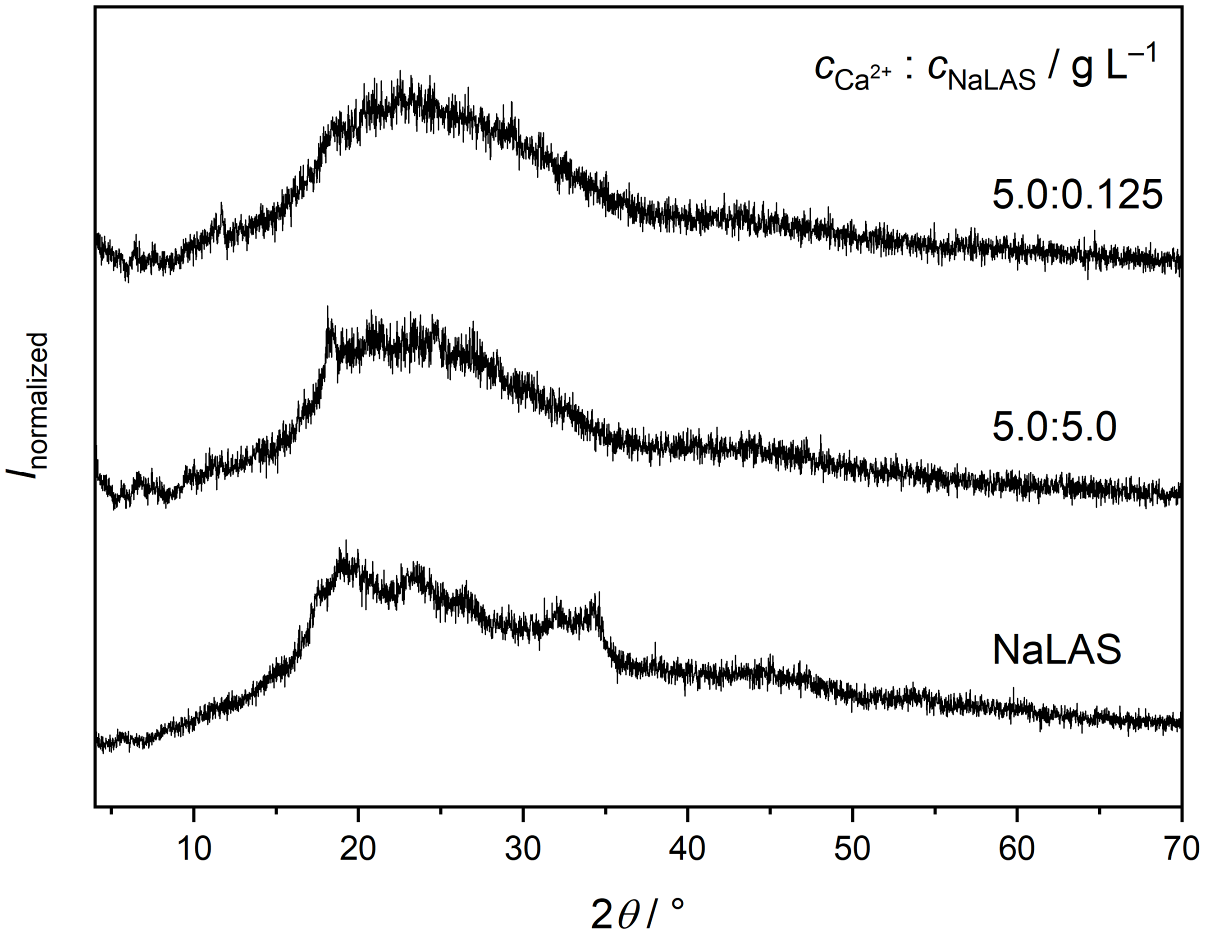

Figure S4 Powder X-ray diffractograms of sodium linear alkylbenzene sulfonate (NaLAS) and precipitates formed by adding $CaCl_2$ to surfactant solutions, at two different Ca^{2+}:NaLAS weight ratios. The intensities were normalized such that the highest value in each diffractogram is 1.00.

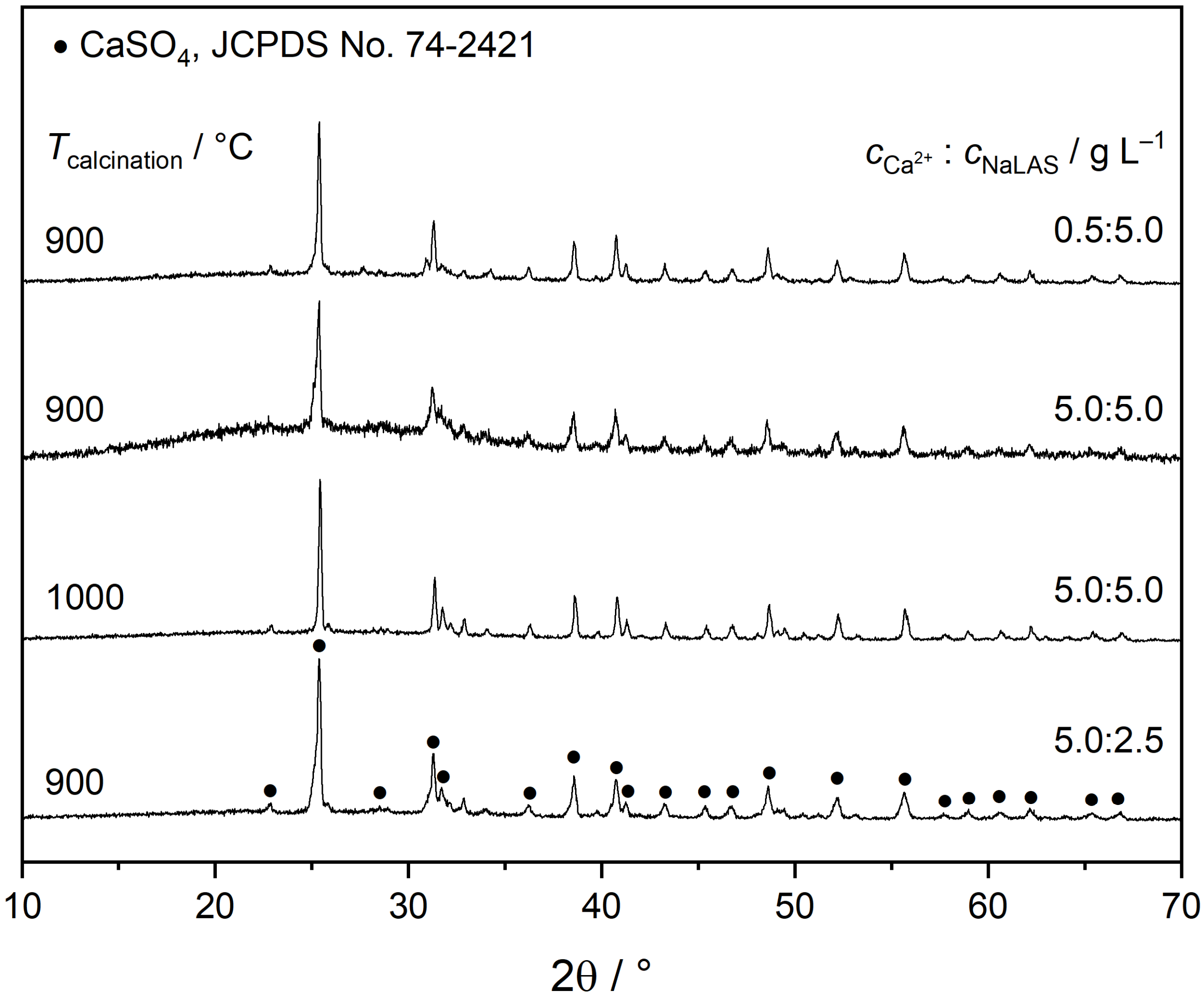

Figure S5 Powder X-ray diffractograms of calcium linear alkylbenzene sulfonate ($CaLAS_2$) precipitates, calcined at 900 °C (24 hours) or 1000 °C (16 hours). The starting solids formed in the mixtures of NaLAS and $CaCl_2$ at two different Ca^{2+}:NaLAS weight ratios. The intensities were normalized such that the highest value in each diffractogram is 1.00.

Table S1 Concentration of sodium linear alkylbenzene sulfonate (NaLAS) and Ca^{2+} in surfactant – $CaCl_2$ dispersions, the atomic percentages of Na^+ and Ca^{2+}, as well as the Na^+:Ca^{2+} molar ratios (with standard deviation) in the $CaLAS_2$ precipitates forming in the dispersions.

c_{NaLAS} / g L^{-1}	$c_{Ca^{2+}}$ / g L^{-1}	atom%$_{Na^+}$	atom%$_{Ca^{2+}}$	n_{Na^+} / $n_{Ca^{2+}}$
2.50	5.00	0.5 ± 0.5	34.9 ± 2.1	0.01 ± 0.01
5.00	0.50	3.9 ± 1.8	34.6 ± 2.3	0.1 ± 0.1
5.00	1.00	0.4 ± 0.4	34.9 ± 4.9	0.01 ± 0.01
5.00	2.00	1.8 ± 0.8	37.8 ± 3.0	0.05 ± 0.02
5.00	3.00	1.4 ± 0.7	34.7 ± 1.5	0.04 ± 0.02
5.00	4.00	0.8 ± 0.6	36.8 ± 0.8	0.02 ± 0.02
